# Development and validation of clinical prediction models to risk stratify patients presenting with small pulmonary nodules: a research protocol

**DOI:** 10.1186/s41512-018-0044-3

**Published:** 2018-11-29

**Authors:** Jason L. Oke, Lyndsey C. Pickup, Jérôme Declerck, Matthew E. Callister, David Baldwin, Jennifer Gustafson, Heiko Peschl, Sarim Ather, Maria Tsakok, Alan Exell, Fergus Gleeson

**Affiliations:** 1 Nuffield Department of Primary Care Health Sciences, University of Oxford, Woodstock Road, OX2 6GG, Oxford, UK; 2 Oxford University Hospitals NHS Foundation Trust, Oxford, Oxford, UK; 3 St James’ University Hospital, Leeds, UK; 4 Nottingham University Hospitals, Nottingham, UK; 5 Optellum Ltd, Oxford, UK

**Keywords:** Lung cancer, Pulmonary nodules, Clinical prediction model

## Abstract

**Introduction:**

Lung cancer is a common cancer, with over 1.3 million cases worldwide each year. Early diagnosis using computed tomography (CT) screening has been shown to reduce mortality but also detect non-malignant nodules that require follow-up scanning or alternative methods of investigation. Practical and accurate tools that can predict the probability that a lung nodule is benign or malignant will help reduce costs and the risk of morbidity and mortality associated with lung cancer.

**Methods:**

Retrospectively collected data from 1500 patients with pulmonary nodule(s) of up to 15 mm detected on routinely performed CT chest scans aged 18 years old or older from three academic centres in the UK will be used to to develop risk stratification models. Radiological, clinical and patient characteristics will be combined in multivariable logistic regression models to predict nodule malignancy. Data from over 1000 participants recruited in a prospective phase of the study will be used to evaluate model performance. Discrimination, calibration and clinical utility measures will be presented.

## Introduction

Small pulmonary nodules are a common finding on computed tomographic (CT) scans of the chest. Up to 75*%* of smokers scanned either as part of their clinical care or in lung cancer screening trials have sub-centimetre pulmonary nodules detected. The US National Lung Screening Trial (NLST) showed that up to 95*%* of lung nodules detected on CT scans of the chest were not malignant. The detection of nodules that are eventually proven to be benign may be expensive and both resource and time consuming with potential associated patient morbidity and mortality.

Current recommendations from internationally accepted Fleischner guidelines [1] and British Thoracic Society (BTS) guidelines for the investigation and management of pulmonary nodules [2] suggests surveillance with CT for nodules of indeterminate risk (see Table 1).

**Table 1:** Abridged guideline recommendations following detection of incidentally detected lung nodules

Nodule diameter and volume	Recommended action
Fleischner guideline [1]	
<6 mm or 100 mm^3^	No routine follow-up if considered low risk or optional CT at 12 months if considered high risk (based on all relevant risk factors
6–8mm or 100–250mm^3^	Surveillance with CT
>8 mm and 250 mm^3^	CT, PET/CT or tissue sampling
British Thoracic Society guideline [2]	
<5 mm or 80 mm^3^	Discharge
5−8 mm or 80−300 mm^3^	Surveillance with CT
>8 mm and 300 mm^3^	Assess using Brock model and surveillance with CT if risk <10*%* or PET-CT if risk ≥10*%*

A substantial proportion of pulmonary nodules detected on CT are judged to have an indeterminate risk of malignancy (≈50*%*) but most (≈97*%*) will be benign. Risk stratification tools that incorporate the age of the patient, their smoking history and their respiratory health could assist clinical decision making, reduce unnecessary investigations and quickly identify those at higher risk.

Existing nodule prediction models have been developed in highly selected patient groups with high rates of malignancy and give very different estimates of risk for smaller nodules. To date, five studies have derived composite prediction models based on a combination of clinical and radiological factors using multivariable logistic regression analysis [2]: Swensen (US, Mayo clinic model) [3], Gould (US, VA model) [4], Li (China) [5], Yonemori [6] (Japan) and McWilliams (Canada, Brock model) [7]. The study by Herder (Netherlands) et al. extended the Mayo clinic model to include positron emission tomography CT (PET-CT) results [8]. There are weaknesses in each of these predictive models particularly with respect to prediction of risk for small nodules, and their generalisability and evaluation. The Brock model was derived from a screening cohort of mostly current or former smokers. The VA model participants were mostly male smokers, and the Mayo cohort were a single cohort managed in the 1980s. The percentage of nodules that were malignant in the Yonemori, Li, VA and Mayo cohorts (75*%* [6], 62*%* [5], 54% [4] and 23*%* [3]) are unrepresentative of the risk in the wider population of patients with pulmonary nodules [9]. Validation of these models in a UK population extends to a single study in 263 patients identified from the lung cancer multidisciplinary team meeting and a nodule follow-up clinic between 2008 and 2013 [10]. Whilst these models discriminate well (C-statistic ranging from 73.5 to 91.2*%* [10]) they differ considerably in their estimate of risk for smaller nodules. For example, a 4 mm upper lobe nodule in a 70-year-old female smoker has a probability of malignancy of 0.3*%* according to the Brock model, 12*%* using the Mayo prediction model and 39*%* according the VA model [2].

This study aims to develop a clinical prediction model which will improve the accuracy of stratifying sub-centimetre lung nodules detected on chest CT scans. The study will incorporate solid nodules of 5 to 15 mm in diameter and aims improve the accuracy of stratifying lung nodules detected on chest CT scans across a wide variety of scanner types, imaging protocols and patient populations. We hypothesise that further characterisation of sub-centimetre pulmonary nodules on chest CT scans will allow us, along with clinical profiling, to improve the accuracy of stratifying lung nodules as benign or malignant, and help guide their management. This will reduce the number of unnecessary investigations for benign nodules and may improve the ability to diagnose—or to instigate investigations that will diagnose malignant nodules earlier than is currently possible.

## Methods

### Sources of data

The development and validation of the risk stratification models for pulmonary nodules will be done using data from the artificial intelligence (AI) and Big Data for Early Lung Cancer Diagnosis (IDEAL) study. IDEAL is a National Institute for Health Research (NIHR) Invention for Innovation (i4i) funded project to build and evaluate a new computer-aided prediction model for malignancy in small pulmonarynodules.

#### Study design

The IDEAL study consists of two phases: a retrospective data collection phase and a prospective study. The risk stratification models will be derived using retrospectively collected data from the phase 1 of IDEAL and evaluated using prospective data collected in the phase 2. The model development team has access to clinical patient data, and to simple clinical observations about the CTs, made by radiologists. There is also an AI imaging model under independent development; this is trained using only data from CT images, with no patient data or human-derived CT observations.

#### Data extraction

CT chest scans reported as containing pulmonary nodules will be identified by a thoracic radiologist reporting the scan, or through an electronic search of CT chest scans previously performed on patients as part of their routine clinical care in the study sites. The scans will be anonymised prior to analysis.

#### Key study dates

The retrospective data collection phase began in January 2018 and is ongoing. The prospective study is expected to start in August 2018 and will end the completion of the last patient’s follow-up (August 2020).

### Participants

IDEAL is collecting data from three academic centres or *partners*; these are as follows:

- Oxford University Hospital NHS Foundation Trust
- Leeds Teaching Hospital NHS Trust
- Nottingham University Hospitals NHS Trust

Each of the three IDEAL partners is expected to contribute 500 patients to the phase 1 of the trial and at least 350 patients for phase 2 (see “Sample size” section for justification of sample size)

#### Inclusion and exclusion criteria

Inclusion criteria are the same for the phase 1 and phase 2 of IDEAL. A patient is eligible for inclusion in the study if they are as follows:

- Male or female, aged 18 years or above.
- Reported as having pulmonary nodule(s) of 5–15 mm detected on CT chest scan
- CT slice thickness of 3 mm or less.

The patient will *not* be included in the study if *any* of the following apply:

- Patient has more than 5 nodules of at least 5 mm.
- Technically inadequate CT scan (see Appendix for details).
- Diagnosis is unknown or could not be established.
- Current or prior history of malignancy in the last 5 years.

### Outcome

The outcome or *ground truth* for each nodule will be established routinely in clinical care using the accepted published standards of the following:

- Histology
- 1 year for volume stability or 2 years for diameter stability, for benign nodules only
- Expert opinion, for subpleural or perifissural lymph nodes only
- Nodule resolution (i.e. infection clears up)

Benign nodules will be coded as zero, malignant nodules as 1.

### Predictors

The following radiographic and clinical variables are available for inclusion into the risk stratification models. These have been selected because they either have been shown to be associated with the risk of nodule malignancy or benignity or have been used in other nodule prediction models.

We anticipate non-response to be an issue for variables such as “year when stopped smoking”, “smoking pack years” and “known industrial exposure” which may preclude their inclusion into the models (see the “Handling missing data” section for details of our method to handle missing data).

### Sample size

A key concept in the consideration of the sample size for clinical prediction models for binary outcomes is the number of events per variable (EPV) [11]. The number of EPV is the number of events divided by the degrees of freedom considered in developing the prediction models. Roughly 10 EPVs have been proposed for accurate estimation of regression coefficients in a logistic regression model [12], whereas a minimum of 20 EPV are required to minimise differences between the bootstrap-corrected estimates and independent validation [11]. This implies that with a sample of 1500 at a prevalence of 10%, models with up to 15 degrees of freedom (df) can be accommodated and allow for accurate estimation of regression coefficients but may not ensure minimal differences between the performance of the models in the derivation and evaluation stage. Previous lung nodule risk models have required a small number of variables. For example, the full Brock model (2b) [7] used 12 df, Mayo clinic model [3] 7 df and VA model [4] 4 df.

### Statistical analysis methods

We intend to build two clinical prediction models. A “full” model including all of the variables listed in Table 2 subject to the missing data criteria outlined previously, and a parsimonious model using a backwards selection criteria to drop variables that are not independently prognostic for malignancy.

**Table 2:** Candidate predictors

Radiographic	Clinical or patient characteristic
Number of nodules annotated	Age at CT scan
Does patient have other relevant nodules	Sex
Were there earlier scans reported negative	Smoking status
Nodule location	Number smoking pack years
Is nodule shape affected by emphysema	Year when stopped smoking
Maximum diameter measured on a 2d axial	Known COPD or emphysema
CT slice	Known coronary artery calcification
	Known pulmonary fibrosis
	Known industrial exposure
	Family history lung cancer

#### Handling of predictors

We will assess whether any continuous predictors in the full-model exhibit a non-linear relationship with the risk of malignancy. In particular, we will carefully check for non-linearity of nodule size against risk, as the Brock model found it necessary to compensate for this [7]. Non-linear variables will be modelled using fractional polynomials where appropriate [13].

#### Model-building procedure

Regression coefficients for both models will be estimated using maximum likelihood estimation in a logistic regression model. The open-source statistical software R [14] will be used with the *glm* function to estimate coefficients.

#### Handling missing data

For some of the clinical variables (such as family history of cancer), we anticipate missing data. To ensure generalisability and prevent loss of efficiency, we will explore methods for handling missing data either by creating a “missing” level for a factor or by using multiple imputation with chained equations (mice) [15]. This will ensure that we can utilise nodules with partially observed clinical data. We will only consider imputation for variables with less than 50% (across the whole cohort) of the data is missing, and we are confident that the missingness pattern can be considered “random” conditioning on the predictor variables in the models. Multiple imputation is considered valid under assumptions that the data are missing at random (MAR) dependent on the observed variables [16] but this assumption cannot be tested. The multiple imputation process will create *m* data sets (m to be determined later but likely to be between 10 and 50) and *m* models and *m* sets of parameter estimates. Parameter estimates for the final models will be combined using Rubin’s rules [17].

#### Internal validation

We will assess the *out of sample* performance of the models using bootstrap-based methods. This entails estimating the apparent performance of the models in the dataset used for development and then repeatedly drawing bootstrap samples (resampling with replacement) and re-estimating the models in order to obtain estimates of model-optimism. This is then subtracted from the apparent performance measure to obtain a optimism-correct performance measure [18].

#### Measures used to assess model performance

Discrimination will be summarised using the c-statistic (equivalent to the AUC) with 95*%* confidence intervals. Calibration of the models will be summarised by the intercept and slope of the validation curve. A calibration plot contrasting predicted probabilities with observed probabilities will be presented.

#### Identification of risk groups

Risk groups will be identified using false negative criteria of 0*%*, 0.5*%* and 1*%*. A false negative criteria has been selected because the prediction rule needs to have good *rule out* properties (high sensitivity). Using the predicted risk scores from the final model equations, we will determine the thresholds which attain the desired false negative rate for the minimum number of false positives. As currently all indeterminate nodules are followed up, the true negatives rate represents the potential reduction in unnecessary investigations.

### Prospective evaluation of the model

#### Missing data

We do not expect the same level of missing covariate data from the prospective data as in the retrospective data used for the model, and so, we will use only complete data for the validation process. If missing data exceeds 20*%*, then sensitivity analyses will be performed on a data set in which the missing covariate data set has been imputed as per the development stage.

### Reporting

We will closely follow the TRIPOD guideline for transparent reporting of multivariable prediction models [19] and produce the following results from the model development stage.

- Diagram showing the flow of participants through the study, including the number of participants, with and without the outcome.
- Table of patient characteristics (demographic, clinical features and radiologic variables—including all candidate predictors in the models). Includes level of missing data per variable.
- Reporting of the unadjusted association between each candidate predictor and the outcome.
- Table of coefficient estimates (as beta coefficients and odds ratio) with confidence intervals.
- The prediction equation in full, with sufficient detail so that individual predictions can be made.
- Discrimination will be summarised using the c-statistic (equivalent to the AUC) with 95*%* confidence intervals.
- Intercept of validation curve (with perfect calibration the value would be 0).
- Slope of validation curve (with perfect calibration, the slope would be 1).
- A calibration plot
- False negative and false positive rates with 95*%* confidence intervals for the three decision thresholds defined in the model development stage.

## Discussion

This protocol describes the methods and statistical analysis plan to develop and evaluate clinical prediction models for pulmonary nodules. Previous prediction models for lung nodules have been based on highly selected patient groups with high rates of malignancy and give very different estimates of risk for smaller nodules. A robustly developed and validated clinical prediction model which generalises to a wide range of patients seen in clinical practice is highly desirable.

## Appendix

### Definition of technically inadequate CT

1. The series should have <=3 mm slice thickness to be acceptable for markup. In practice, it is expected that scans with <=2 mm are available and they are preferred to 3 mm thick scans.
2. The CT should not be from a tilted CT acquisition.
3. The CT should be free from artefacts which would affect the appearance of the nodule (motion) or the capacity of the clinician to make a diagnosis on the clinical image (noise). Such artefacts could manifest as follows:
  Shifting structures in consecutive slices, which would be particularly visible in coronal or sagittal slices
  Respiratory or cardiac motion
  Excessive noise level that affects the nodule appearance.

## Abbreviations

AI: Artificial intelligence
AUC: Area under the curve
BTS: British thoracic society
COPD: Chronic obstructive pulmonary disease
CT: Computed tomography
Df: Degrees of Feedom
EPV: Evens per variable
MAR: Missing at random
NIHR: National institute for health research
NHS: National health service
NLST: National lung screening trial
PET-CT: Positron emission tomography computed tomography
UK: United Kingdom
VA: Veterans affairs
